# Combining plasma phospho-tau and accessible measures to evaluate progression to Alzheimer’s dementia in mild cognitive impairment patients

**DOI:** 10.1186/s13195-022-00990-0

**Published:** 2022-03-29

**Authors:** Alexa Pichet Binette, Sebastian Palmqvist, Divya Bali, Gill Farrar, Christopher J. Buckley, David A. Wolk, Henrik Zetterberg, Kaj Blennow, Shorena Janelidze, Oskar Hansson

**Affiliations:** 1 Clinical Memory Research Unit, Faculty of Medicine, Lund University, Lund, Sweden; 2 Memory Clinic, Skåne University Hospital, SE-20502 Malmö, Sweden; 3 GE Healthcare, Chalfont St Giles, UK; 4 Department of Neurology, Penn Memory Center, University of Pennsylvania, Philadelphia, PA USA; 5 Department of Psychiatry and Neurochemistry, the Sahlgrenska Academy at the University of Gothenburg, Mölndal, Sweden; 6 Clinical Neurochemistry Laboratory, Sahlgrenska University Hospital, Mölndal, Sweden; 7 Department of Neurodegenerative Disease, UCL Institute of Neurology, Queen Square, London, UK; 8 UK Dementia Research Institute at UCL, London, UK; 9 Hong Kong Center for Neurodegenerative Diseases, Hong Kong, China

**Keywords:** Plasma biomarkers, Alzheimer’s disease, p-tau, Mild cognitive impairment, Dementia

## Abstract

**Background:**

Up to now, there are no clinically available minimally invasive biomarkers to accurately identify mild cognitive impairment (MCI) patients who are at greater risk to progress to Alzheimer’s disease (AD) dementia. The recent advent of blood-based markers opens the door for more accessible biomarkers. We aimed to identify which combinations of AD related plasma biomarkers and other easily accessible assessments best predict progression to AD dementia in patients with mild cognitive impairment (MCI).

**Methods:**

We included patients with amnestic MCI (*n* = 110) followed prospectively over 3 years to assess clinical status. Baseline plasma biomarkers (amyloid-β 42/40, phosphorylated tau217 [p-tau217], neurofilament light and glial fibrillary acidic protein), hippocampal volume, *APOE* genotype, and cognitive tests were available. Logistic regressions with conversion to amyloid-positive AD dementia within 3 years as outcome was used to evaluate the performance of different biomarkers measured at baseline, used alone or in combination. The first analyses included only the plasma biomarkers to determine the ones most related to AD dementia conversion. Second, hippocampal volume, *APOE* genotype and a brief cognitive composite score (mPACC) were combined with the best plasma biomarker.

**Results:**

Of all plasma biomarker combinations, p-tau217 alone had the best performance for discriminating progression to AD dementia vs all other combinations (AUC 0.84, 95% CI 0.75–0.93). Next, combining p-tau217 with hippocampal volume, cognition, and *APOE* genotype provided the best discrimination between MCI progressors vs. non-progressors (AUC 0.89, 0.82–0.95). Across the few best models combining different markers, p-tau217 and cognition were consistently the main contributors. The most parsimonious model including p-tau217 and cognition had a similar model fit, but a slightly lower AUC (0.87, 0.79–0.95, *p* = 0.07).

**Conclusion:**

We identified that combining plasma p-tau217 and a brief cognitive composite score was strongly related to greater risk of progression to AD dementia in MCI patients, suggesting that these measures could be key components of future prognostic algorithms for early AD.

**Trial registration:**

NCT01028053, registered December 9, 2009.

## Background

Prodromal Alzheimer’s disease (AD) is a common cause of mild cognitive impairment (MCI), but it is very difficult to clinically differentiate it from other causes of MCI [1]. Generally, between 20 to 40% of MCI patients will progress to AD dementia within a few years [2, 3]. Improving the identification of patients at greater risk of further cognitive decline is thus important for clinical practice for patients and families, in clinical trials to enroll patients having AD pathology, and in the future for selecting patients for treatment with disease-modifying drugs. The key protein causing AD, beta-amyloid (Aβ) and tau, can be measured either in the cerebrospinal fluid (CSF) with lumbar puncture or in the brain with positron emission tomography (PET). These can be used to help in determining the risk of progression to AD dementia in individuals with MCI [4–7]. However, CSF collection may be regarded as invasive and PET scans are costly and have limited availability, which hampers the use of these methodologies in clinical practice from a global perspective. With the recent advent of blood-based biomarkers, we can now measure a variety of proteins related to AD in a time- and cost-effective manner and investigate how well such markers can inform disease diagnosis and prognosis [6]. The molecular pathways that can be investigated with plasma biomarkers also now extend beyond Aβ and tau. These include for example neurodegenerative markers such as neurofilament light (NfL) and glial activation biomarkers such as glial fibrillary acidic protein (GFAP) [6, 8]. Very promising results in non-demented patients suggest that plasma tau phosphorylated at threonine 217 (p-tau217), in combination with cognitive performance and apolipoprotein E (*APOE*) genotype, was the best marker to predict conversion to AD dementia within 4 years, with very high accuracy [1]. These recent results were derived from only two cohorts, and thus, we still need to validate the optimal markers of conversion in other independent cohorts and determine the most consistent results before we can implement such prognostic algorithms in clinical practice globally.

The current study focuses on a subset of amnestic MCI patients previously enrolled in a 3-year clinical trial, which was originally designed to determine the accuracy of [^18^F]flutemetamol PET to predict subsequent conversion to dementia [7]. We now investigated which combinations of key plasma biomarkers and other commonly used and accessible markers of AD were related to progression to AD dementia. First, we studied the accuracy of plasma biomarkers to identify MCI patients who are likely to progress to AD dementia. In this cohort, we quantified four plasma biomarkers: p-tau217, the ratio of Aβ42/Aβ40, as well as NfL and GFAP. Next, we considered whether combining the best performing plasma biomarkers with hippocampal volume, *APOE* genotype, and a composite cognitive score would further improve the discrimination between MCI patients who progressed to AD dementia and those who did not.

## Methods

### Participants

Patients for this study were originally included from a completed clinical trial that aimed at investigating the efficacy of [^18^F]flutemetamol Aβ-PET to predict conversion from MCI to probable AD dementia (NCT01028053, 2009-2014). All participants had amnestic MCI based on the Petersen and Morris criteria [9], a Clinical Dementia Rating (CDR) of 0.5, were 60 years or older, had a Mini-Mental State Examination (MMSE) between 24 and 30, and a score on the Modified Hachinski Ischemic Scale equal or less to 4. The main exclusion criteria were other significant neurological or psychiatric conditions. Participants and trial outcomes have been described in greater details previously [7]. The present study included a subset of the initial trial sample, namely participants who had available plasma for analysis, resulting in 110 of the original 232 participants.

### Outcome

Participants underwent evaluation by trained personnel at each site that consisted of neuropsychological tests as well as the CDR, MMSE, and activities of daily living every 6 months for up to 36 months. After each visit, participant data was reviewed by members of the clinical adjudication committee, who were blinded to the biomarker data, to determine clinical diagnosis. Diagnosis of probable AD was based on the National Institute of Neurological and Communicative Disorders and Stroke–Alzheimer’s Disease and Related Disorders Association (NINCDS-ADRDA) criteria [10]. For the present study, the clinical outcome was conversion to AD dementia over 36 months. Only those that were classified as probable AD dementia (according to the NINCDS-ADRDA criteria) and had a positive Aβ-PET at baseline were coded as progressors to AD dementia (in accordance with the NIA-AA definition of AD [11]). Aβ-positivity was defined based on a predefined threshold of 1.56 standardized uptake value ratio from a global [^18^F]flutemetamol Aβ-PET region including precuneus, cingulate, frontal, and lateral temporal regions [7]. Those who remained MCI (*n* = 71) and those who were given a clinical diagnosis of dementia but were Aβ-PET negative (*n* = 13) were coded as non-progressors to AD dementia, hereafter refer to as non-progressors.

### Plasma biomarkers

Plasma p-tau217 and NfL concentrations were measured at Lund University, Sweden, for all participants. P-tau217 was measured using an immunoassay on the Meso-Scale Discovery (MSD) platform developed by Eli Lilly as described previously [12]. NfL was measured using the commercially available Simoa immunoassay [13, 14]. Plasma Aβ40, Aβ42, and GFAP concentrations were measured at the Clinical Neurochemistry Laboratory, University of Gothenburg, Sweden, in 80 participants out of 110, those with enough plasma left. These three proteins were measured using the Simoa Human Neurology 4-Plex E (N4PE) assay (Quanterix®, Billerica, MA, USA).

### Cognitive tests

Participants underwent different neuropsychological tests as part of the clinical evaluations. For this study, given the smaller sample size, we focused on a composite measure focusing on cognitive domains affected early in AD, rather than on multiple individual tests. Our measure of interest was a modified version of the Preclinical Alzheimer’s Cognitive Composite 5 (PACC) score [15]. The tests included in the modified PACC (mPACC) were the MMSE, Logical Memory Scale II delayed recall, Digit Symbol Substitution Test, Category Fluency of animals, and vegetables (the sum of both categories formed the Category Fluency score). The original PACC includes two measures of memory recall (Logical Memory and the Free and Cued Selective Reminding Test); however, because only one was available, Logical Memory delayed recall was given twice the weight to maintain the same proportion of memory as in the original composite score as done previously [16]. All tests were *z*-scored based on the current sample and then averaged to generate the mPACC used in statistical analyses.

### Other predictors

In line with the original clinical trial and considering important factors related to AD etiology, we also included *APOE* genotype and hippocampal volume in analyses. *APOE* genotype was available for 100 out or 110 participants. People with at least one *ε*4 allele were considered *APOE*4 carriers. Structural T1-weighted magnetic resonance imaging was also acquired at baseline from which the hippocampus was segmented using a local, patch- based label fusion approach [17]. Hippocampal volume was then adjusted for total intracranial volume using a scaling factor related to the difference between individual subject and MNI152 template space. More details have been described in Wolk et al. [7].

### Statistical analysis

All analyses were performed using R version 4.0.5. Demographics, plasma biomarkers, and markers of interest were compared between patients with MCI who progressed to AD dementia vs. those who did not progress to AD dementia using *t*-test or chi-square. The main analyses were then logistic regressions to determine which combinations of markers best discriminated progressors to AD dementia from non-progressors. Variables of interest were *z*-scored prior to the logistic regressions, so that odds ratio between variables and models are easily comparable. We used the Multi-Model-Inference R package version 1.43.17 that generates models with the best combinations of biomarker and the pROC package version 1.17.0.1 to compare them to one another. Models were ranked based on model fit using corrected Akaike Information Criteria (AICc), appropriate for smaller sample sizes, where lower values denote better model fit. The model with the lowest AICc represented the best model fit and was compared to subsequent models with the goal to retain the most parsimonious models. A change in AICc lower than 2 between models implied that the two models had a similar fit. ANOVA was also used to compare the best model to subsequent models. Area under the curve (AUC) and its 95% confidence interval computed from the DeLong method were also calculated for each model, and AUCs between models were also compared with the DeLong method. This approach using multi-model inference to retain the most discriminant markers has been validated recently in two independent cohorts [1].

We performed two sets of logistic regression analyses. Given that only 80 out of the 110 participants had all plasma biomarkers level (p-tau217, NfL, Aβ42/Aβ40, and GFAP), we first aimed at identifying which plasma biomarker(s) were most related to progression to AD dementia. In this first set of model comparisons, only the four plasma biomarkers were entered as predictors, with conversion to AD dementia as the outcome. Plasma biomarkers with odds ratios with a *p*-value < 0.10 were kept for further analysis. Second, the same approach of using AICc for model selection was repeated to distinguish among possible models combining the key identified plasma biomarker(s), *APOE*4 status, hippocampal volume, and mPACC with conversion to AD dementia as outcome. The overall goal was to determine the best (lowest AICc) and the most parsimonious models (similar fit and AUC as the best model) combining plasma and key AD markers in relation to clinical progression. Sensitivity analyses also included basic demographics (age, sex, and education) as additional predictors to assess whether they were important factors in assessing risk of progressing to AD dementia.

### Data availability

Anonymized data can be shared to qualified academic researchers after request for the purpose of replicating procedures and results presented in the study. Data transfer must be in agreement with EU legislation regarding general data protection regulation and decisions by the Ethical Review Board of Sweden and Region Skåne, which should be regulated in a data transfer agreement.

## Results

### Participants

**Fig. 1:**
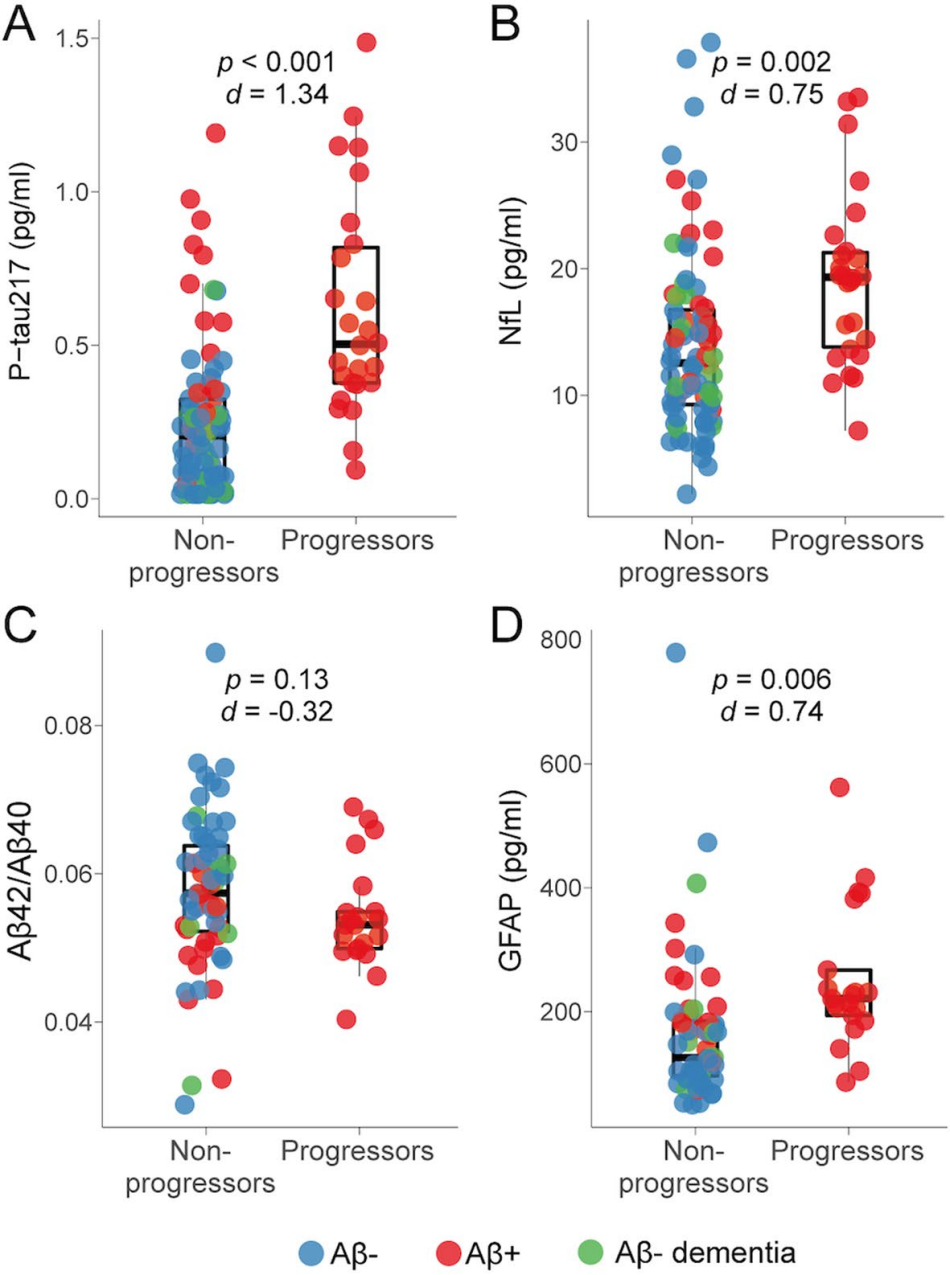
Comparisons of plasma biomarkers between MCI who progressed to AD dementia and those who did not. Levels of plasma p-tau217 (**A**), NfL (**B**), Aβ42/Aβ40 ratio (**C**), and GFAP (**D**) between MCI patients who did not progress to AD dementia (non-progressors) vs. those who progressed to AD dementia (progressors) within 3 years. Boxes represent the first and third quartile of each distribution, and whiskers extend up to 1.5-times the interquartile range. Corresponding *p*-value and Cohen’s *d* effect size are reported on the top of each panel. Aβ, beta-amyloid; GFAP, glial fibrillary acidic protein; NfL, neurofilament light; MCI, mild cognitive impairment; p-tau217, phosphorylated tau 217

The sample of this study, i.e*.*, MCI participants with available plasma samples (*n* = 110), was comparable to the whole sample enrolled in the initial clinical trial (*n* = 232). The current sample did not differ from the whole clinical trial sample in terms of demographics, hippocampal volume, or mPACC score (*p*-values between 0.33 and 0.95). In this study, we focused on conversion to AD dementia over 3 years as the clinical outcome, which corresponds to having received a clinical diagnosis of probable AD dementia (according to the NINCDS-ADRDA criteria) and being Aβ-positive based on [^18^F]flutemetamol. We did not focus on predicting amnestic dementia of non-AD type, given that those individuals will not be suitable for anti-Aβ and anti-tau therapies. In the whole trial, 22% (52/232) converted to AD dementia within the 3-year observation period and 24% (26/110) in the current sample. Of note, 13 participants developed Aβ-negative dementia. Patients who progressed to AD dementia differed on three out of the four plasma biomarkers of interest compared to the non-progressors to AD: they had higher baseline levels of p-tau217, NfL, and GFAP, but similar levels of the Aβ42/Aβ40 ratio (Fig. 1). The biggest difference between groups was on p-tau217, with a large effect size, while NfL and GFAP had a moderate effect size (Cohen’s *d* on Fig. 1). MCI progressors did not differ from non-progressors to AD on age, sex, and education, but had a greater proportion of *APOE*4 carriers, lower hippocampal volume, and cognitive performance (Table 1). In either progressors or non-progressors, baseline plasma biomarkers levels were not associated with baseline mPACC score (all *p*-values > 0.32).

**Table 1:** Demographics, plasma biomarkers and clinical variables

	Non-progression to AD dementia (*n* = 84)	Progression to AD dementia (*n* = 26)	***p***-value
Age, years	71.52 ± 8.20	74.77 ± 8.12	0.08
Sex F to M (%)	38:46 (45%)	14:12 (54%)	0.59
Years of education	13.62 ± 4.01	14.42 ± 3.81	0.36
*APOE*4 carriers: non carriers (%)^a^	21:56 (27%)	14:9 (61%)	0.006
Plasma p-tau217, pg/ml	0.25 ± 0.24	0.62 ± 0.36	< 0.001
Plasma NfL, pg/ml	13.94 ± 6.99	19.14 ± 6.77	0.002
Plasma Aβ42/Aβ40^b^	0.06 ± 0.01	0.05 ± 0.01	0.13
Plasma GFAP^b^, pg/ml	164.69 ± 117.31	251.30 ± 115.21	0.006
Hippocampal volume, cm^3^	4.72 ± 0.72	4.41 ± 0.68	< 0.001
mPACC	0.15 ± 0.82	− 0.50 ± 0.82	0.001

Data are presented as mean ± standard deviation unless specified otherwise. *p*-values were obtained from *t*-test or chi-square (sex and *APOE*4) comparing the two MCI groups, i.e., those who progressed to AD dementia within three years vs. those who did not

*Abbreviations Aβ* beta-amyloid, *APOE*4 apolipoprotein E genotype (carrying at least one ε4 allele), *GFAP* glial fibrillary acidic protein, *mPACC* modified Preclinical Alzheimer’s Cognitive Composite, *NfL* neurofilament light, *p-tau217* phosphorylated tau 217

^a^ Genotype missing for 7 non progressors and 3 progressors

^b^ Values available for 80/110 participants (59 non-progressors and 21 progressors)

### Plasma biomarkers to identify progression to AD dementia

As a first step, we aimed to select the plasma biomarkers that would yield the best discrimination between MCI who progressed to AD dementia from those who did not. These analyses included the 80 participants who had all four plasma biomarkers available (GFAP and Aβ42/Aβ40 not being available for the full sample). The top five models (based on best fit of the model, i.e., lowest AICc), which included different combinations of the four plasma biomarkers are reported in Table 2. The best model included only plasma p-tau217 as a predictor. The subsequent models included combinations of plasma biomarkers, but p-tau217 was the only biomarker kept in all models and was significant in all models. Further, the best model (p-tau217 only) did not have a significantly different model fit or AUC compared with the subsequent ones (all *p*-values > 0.17 from ANOVAs for model fit and all *p*-values > 0.81 from bootstrapping for AUC), understandably so given that the differences in AICc or AUC between all models were minor (less than 2 and less than 0.01 respectively; Table 1). As such, this first step revealed unequivocally that the best plasma biomarker related to conversion to AD dementia in this cohort was p-tau217 and that the other biomarkers had negligible contribution to the models. Only plasma p-tau217 was thus retained for the next set of analyses.

**Table 2:** Association of plasma biomarkers with conversion to AD dementia

***n*** = 80	Model		Odds ratio (***p***-value)			
	AICc	AUC [95% CI]	p-tau217	NfL	GFAP	Aβ42/Aβ40
Model 1 (best model)	76.2	0.840 [0.748, 0.933]	3.11 (0.0002)			
Model 2	76.5	0.843 [0.754, 0.931]	2.78 (0.0009)	1.54 (0.16)		
Model 3	76.9	0.836 [0.745, 0.927]	2.86 (0.0008)		1.44 (0.22)	
Model 4	77.9	0.846 [0.761, 0.931]	2.65 (0.0022)	1.44 (0.26)	1.31 (0.37)	
Model 5	78.3	0.841 [0.748, 0.934]	3.04 (0.0003)			0.90 (0.75)

Results from logistic regression models discriminating MCI patients who progressed to AD dementia within three years (*n* = 21) vs. those who did not (*n* = 59) in the subsample with all plasma biomarkers. Models are ordered based on AICc (lower values representing better model fit) and odds ratio (*p*-value) of each variable included in the corresponding models are reported. Odds ratio values represent the “increased risk” of converting to AD dementia for each increase in standard deviation of the plasma biomarker value. Note that a difference in AICc greater than 2 between models would imply a better fit for the model with the lowest AICc

*Abbreviations*: *Aβ* beta-amyloid, *AICc* corrected Akaike information criteria, *AUC* area under the curve, *GFAP* glial fibrillary acidic protein, *NfL* neurofilament light, *p-tau217* phosphorylated tau 217

To further investigate the value of the different plasma biomarkers, we used the same methodology to assess which ones best related to Aβ-PET positivity at baseline as an outcome instead of clinical progression. The results were very consistent with those related to progression to AD dementia. The best model to evaluate brain amyloidosis included only p-tau217 and had an AUC of 0.90 (95% CI 0.83 to 0.96; AICc = 69.5). The subsequent best models all included p-tau217 with one or two of the other plasma biomarkers, although with very little change in model fits or performances (change in AICc less than 0.5 across the top five models and all AUCs of 0.91). P-tau217 was also always the only significant predictor in the different models (other biomarkers had *p*-values equal or greater than 0.17).

### P-tau217 and other key AD measures to identify progression to AD dementia

**Fig. 2:**
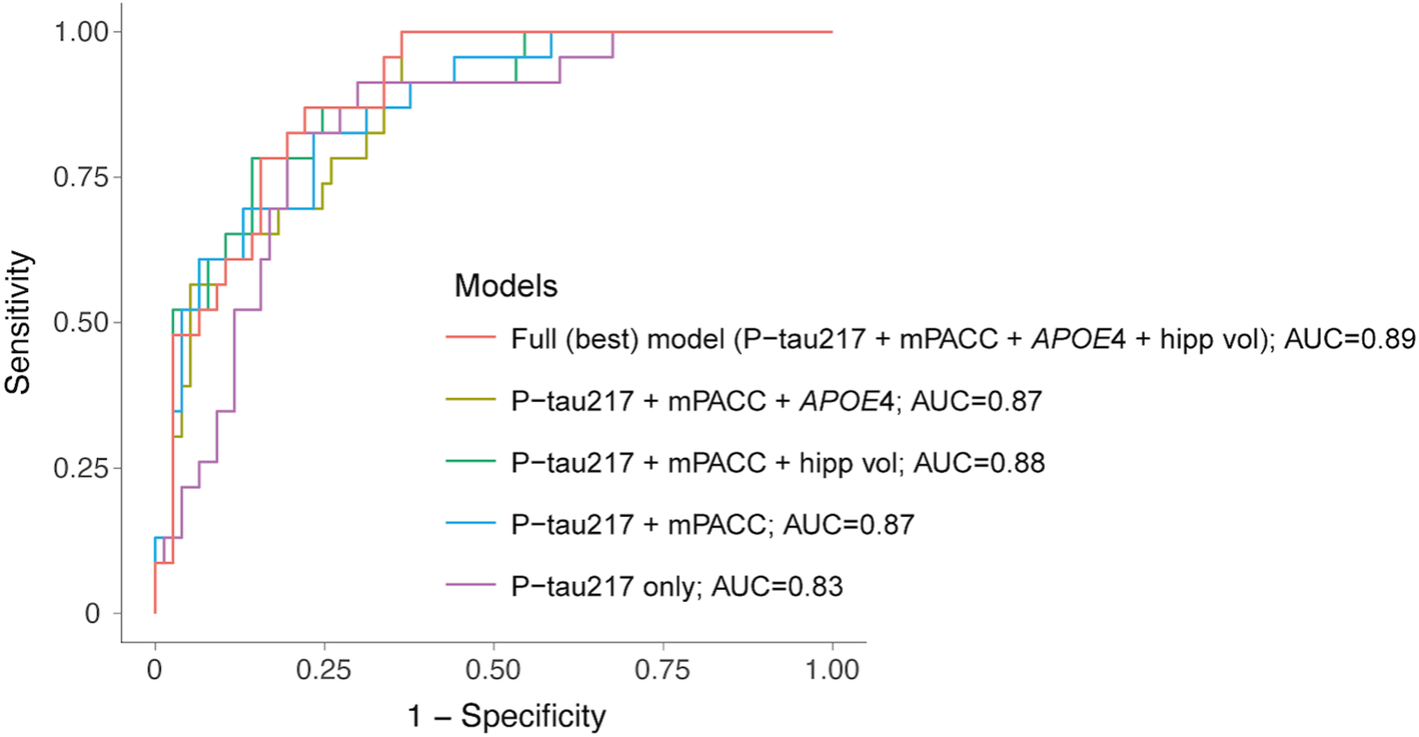
Receiver operating characteristic curves from different combinations of markers related to conversion to AD dementia. Receiver operating characteristic curves from logistic regression models discriminating MCI patients who progressed to AD dementia within 3 years (*n* = 23) vs. those who did not (*n* = 77) in the subsample with plasma p-tau217 and all other AD markers of interest (global cognition from mPACC, hippocampal volume and *APOE*4 status). All details of the different models are reported in Table 3. APOE, apolipoprotein E genotype (carrying at least one ε4 allele); AUC, area under the curve; hipp, hippocampal volume (adjusted for total intracranial volume); mPACC, modified Preclinical Alzheimer’s Cognitive Composite; p-tau217, phosphorylated tau 217

Next, we evaluated whether easily accessible markers of AD combined with p-tau217 could improve identifying conversion to AD dementia. We included mPACC as a composite measure of cognitive performance, *APOE*4 status, and hippocampal volume along with p-tau217 as predictors. We used a similar approach as in the previous step, where combinations of variables were tested and the best models (based on best fit of the model, i.e., lowest AICc) are reported in Table 3. Ten participants did not have *APOE* genotype available, and thus, these analyses included 100 participants. Here, the model with the highest AUC included p-tau217 and all other markers of interest (Fig. 2). The main contributors to the model were p-tau217 and mPACC (odds ratio of 2.25 and 0.43 respectively, all *p*-values ≤ 0.02) while hippocampal volume and APOE contributed at trend level (*p*-values 0.10–0.12). The subsequent best models included progressively fewer AD markers. Models fits (AICc) were similar across the top four models (change in AICc less than 1) indicating similar performance of the models, but the AUC diminished slightly, from 0.89 in the full model (model 1 in Table 3) to 0.87 in the most parsimonious model that included only p-tau217 and mPACC (model 4 in Table 3). The model fit and AUC of the parsimonious model were not significantly lower than the full model (*p* = 0.07 for model fit and *p* = 0.27 for AUC). As a comparison, all four best models were better than p-tau217 alone or the combined three other measures (Table 3).

**Table 3:** Association of plasma p-tau217, cognition, hippocampal volume, and *APOE*4 genotype with conversion to AD dementia

***n*** = 100	Model			Odds ratio (***p***-value)			
	AICc	AUC [95% CI]	Model comparisons	p-tau217	mPACC	Hipp. volume	***APOE***4
Model 1 (Full model)	82.0	0.889 [0.824, 0.954]	1 vs. 2 or 3: *p* = 0.10 1 vs. 4: *p* = 0.07 1 vs. 5: *p* = 0.01 1 vs. ref: *p* = 0.001	2.25 (0.021)	0.43 (0.008)	0.60 (0.12)	2.81 (0.10)
Model 2	82.3	0.868 [0.796, 0.941]	2 vs. ref: *p* = 0.001	2.66(0.005)	0.38 (0.002)		2.76 (0.099)
Model 3	82.5	0.875 [0.798, 0.953]	3 vs. ref: *p* = 0.001	2.71 (0.004)	0.43 (0.009)	0.60 (0.12)	
Model 4	82.9	0.866 [0.787, 0.945]	4 vs. ref: *p* = 0.001	3.22 (0.0007)	0.38 (0.002)		
Model 5	86.4	0.848 [0.768, 0.929]	5 vs. ref: *p* = 0.007		0.42 (0.006)	0.48 (0.017)	4.33 (0.011)
Reference model	91.9	0.831 [0.743, 0.920]	–	3.14 (0.0001)			

Results from logistic regression models discriminating MCI patients who progressed to AD dementia within 3 years (*n* = 23) vs. those who did not (*n* = 77) in the subsample with plasma p-tau217 and all other AD markers of interest (global cognition from mPACC, hippocampal volume and *APOE*4 status). Models are ordered based on AICc (lower values representing better model fit) and odds ratio (*p*-value) of each variable included in the corresponding models are reported. Odds ratio values represent the “increased risk” of converting to AD dementia for each increase in standard deviation of the marker value. Note that a difference in AICc greater than 2 between models would imply a better fit for the model with the lowest AICc. Model including plasma p-tau217 only was included as the reference model. Comparisons between models were performed using ANOVA and *p*-values are reported

*Abbreviations*: *AICc* corrected Akaike information criteria, *APOE*4 apolipoprotein E genotype (carrying at least one ε4 allele), *AUC* area under the curve, *Hipp.* volume hippocampal volume (adjusted for total intracranial volume), *mPACC* modified Preclinical Alzheimer’s Cognitive Composite, *p-tau217* phosphorylated tau 217, *ref* Reference model (p-tau217 only)

### Sensitivity analyses

Given that *APOE*4 status was missing for 10 participants, we repeated the previous analyses using only mPACC and hippocampal volume combined with p-tau217, to ensure that similar results would be found in the full sample (Table 4). Again, the best model included all variables, but the difference was negligible with the model including only p-tau217 and mPACC as predictors: AICc and AUC were virtually unchanged between the two models, both with a difference lower than 0.02 (model comparisons *p* = 0.14, AUC comparisons *p* = 0.22). As a comparison, the full model was significantly better than p-tau217 and hippocampal volume (*p* = 0.01, AICc difference of 4.6). This sensitivity analysis supports the choice of p-tau217 and global cognition as the most parsimonious model to discriminate MCI who progressed to AD dementia vs. those who did not. Further, adding basic demographic variables (age, sex and education) in the parsimonious model of p-tau217 and mPACC did not improve model performance (AICc = 95.1; AUC [95% CI] = 0.87 [0.80, 0.95]; age, sex and education were not significant predictors and had *p*-values > 0.32).

**Table 4:** Association of plasma p-tau217, cognition, and hippocampal volume with conversion to AD dementia

***n*** = 110	Model			Odds ratio (***p***-value)		
	AICc	AUC [95% CI]	Model comparisons	p-tau217	mPACC	Hipp. volume
Full model (model 1)	91.1	0.878 [0.806, 0.951]	1 vs. 2: *p* = 0.14 1 vs. 3: *p* = 0.01 1 vs. ref: *p* = 0.003	3.16 (0.0002)	0.47 (0.013)	0.65 (0.15)
Model 2	91.1	0.862 [0.784, 0.940]	2 vs. ref: *p* = 0.002	3.54 (< 0.0001)	0.43 (0.004)	
Model 3	95.7	0.866 [0.799, 0.933]	3 vs. ref: *p* = 0.024	2.88 (< 0.0001)		0.52 (0.030)
Reference model	98.7	0.842 [0.763, 0.922]	–	3.13 (< 0.0001)		

Results from logistic regression models discriminating MCI patients who progressed to AD dementia within three years (*n* = 26) vs. those who did not (*n* = 84) in the full sample with plasma p-tau217, global cognition from mPACC, and hippocampal volume. Models are ordered based on AICc (lower values representing better model fit) and odds ratio (*p*-value) of each variable included in the corresponding models are reported. Odds ratio values represent the “increased risk” of converting to AD dementia for each increase in standard deviation of the marker value. Note that a difference in AICc of 2 between models would imply a better fit for the model with the lowest AICc. Model including plasma p-tau217 only was included as the reference model. Comparisons between models were performed using ANOVA and *p*-values are reported

*Abbreviations*: *AICc* corrected Akaike information criteria, *AUC* area under the curve, *Hipp.* volume hippocampal volume (adjusted for total intracranial volume), *mPACC* modified Preclinical Alzheimer’s Cognitive Composite, *p-tau217* phosphorylated tau 217, *ref* Reference model (p-tau217 only)

## Discussion

In this study, we investigated which combinations of plasma biomarkers and markers typically used in AD prognosis provided the best discrimination between MCI patients who progressed to AD dementia over 3 years compared to those who did not. Focusing first on four key plasma biomarkers, p-tau217 was the most predictive marker of clinical progression, both when used alone and in combination with the other plasma measures (AUC of 0.84). There was no meaningful improvement of combining p-tau217 with other plasma biomarkers. However, the discrimination between the two MCI groups was further improved when incorporating a score of global cognition, hippocampal volume, and *APOE*4 genotype as predictors along with plasma p-tau217 (AUC of 0.89). Aiming for a parsimonious model that would have a similar model fit as the one with all variables, we found that including only plasma p-tau217 and a global cognitive score yielded comparable results.

Aβ plaques, tau tangles, and neurodegeneration are the core pathophysiological alterations of AD, as conceptualized in the biomarker-driven AT(N) classification [18]. However, other pathophysiological pathways (X) are being investigated as potentially important in AD, resulting in the proposition of new ATX(N) classification, to be able to incorporate and adapt to new biomarkers [19]. One such pathway is neuroinflammation and glial activation, which can be tracked with novel fluid biomarkers like GFAP, YKL40, and TREM2 [20, 21]. We thus applied an ATX(N)-like framework in the MCI cohort to investigate which plasma biomarkers were most related to conversion to AD dementia. We selected the Aβ42/Aβ40 ratio (A), p-tau217 (T), NfL (N), and the increasingly studied astrocytic marker GFAP (X). Using a data-driven approach allowing all combinations of plasma biomarkers to derive the best models with conversion to AD dementia as outcome, it was clear that p-tau217 was consistently the best biomarker to discriminate MCI progressors from non-progressors. In fact, adding other plasma biomarkers in combination with p-tau217 did not result in improved model fit or better discrimination (no change in AICc or AUC, Table 2). This result further adds to the growing literature of plasma p-tau217 (or p-tau181) as important markers to track AD progression [1, 22, 23].

We did not observe added value of plasma Aβ42/Aβ40 when combined with plasma p-tau217 for either clinical progression or Aβ-PET status. However, we cannot exclude that this result is assay-specific, since Aβ42 and Aβ40 were measured using Simoa immunoassays, which have been shown to be less accurate than certain mass spectrometry-based Aβ assays [12]. Still, a recent study where the outcome was brain amyloidosis also found that at the MCI stage, plasma p-tau217 was the best biomarker to identify Aβ-positive participants, with or without plasma Aβ42/Aβ40 (quantified with mass spectrometry-based assay) as an additional predictor [12]. These results also align with the AD pathophysiology, with Aβ proteins starting to change and plateauing earlier in the AD continuum making them less informative for predicting much later cognitive decline, while p-tau continues to increase through the prodromal stage of the disease to the dementia stage, shown both in studies using CSF [24–26] and plasma [27, 28] biomarkers. Accumulating evidence also suggested close relationship between Aβ and GFAP rather than between GFAP and p-tau [29–32]. While plasma Aβ and GFAP might be more closely associated with Aβ pathology in earlier stages of the disease, p-tau is more associated with clinical progression.

With the idea of implementing a cost-effective predictive model, we also focused on combining p-tau217 with other easily accessible measures in AD. We used measures in line with those investigated in the initial clinical trial in combination with Aβ-PET [7], i.e., hippocampal volume, *APOE*4 genotype and a global score of cognition. Adding these three variables with p-tau217 resulted in the best identification of MCI progressors to AD dementia, with an AUC of 0.89. Previous studies with a similar outcome but using CSF or PET biomarkers rather than plasma also often found an added value of such additional non-biomarker measures [33–35]. However, when comparing the best model that included all variables to the best subsequent combinations of variables, we found that p-tau217 and the global cognitive score mPACC were largely comparable to the full model. Model fits were similar, but the AUC was slightly lower (0.89 for full model vs. 0.87 for p-tau217 + mPACC, *p* = 0.07). Across all models combining p-tau217 with other variables, we should note that mPACC was always a significant contributor, while hippocampal volume or *APOE*4 genotype were often at trend-level with *p*-values around 0.1. Our approach for this study and the main results corroborate the findings from a recent large-scale study from our group where p-tau217, memory score, executive function, and *APOE*4 genotype was the best combination to determine conversion to AD-dementia within 4 years in cognitively normal older adults or MCI patients [1]. As with the current study, NfL, structural measures from MRI, and basic demographics only had little influence on model performance. Notably, model accuracy was similar in both studies, with an AUC of 0.91 in the large-scale study and 0.89 here. Overall, across very different datasets, there is converging evidence that plasma p-tau217 in combination with easily accessible AD markers have the highest potential to help detect individuals at risk of progression to dementia. To move the field forward and get closer to implementing the most promising markers more widely in clinical practice, it is important to validate results evaluating risk of conversion to AD in multiple samples. Further, in cases of smaller sample size as the current study where only minor differences between models existed, we propose that p-tau217 and global cognitive score would be sufficient predictors for a parsimonious, most easily accessible model predicting progression to AD dementia.

## Limitations

There are a few limitations to consider to this study. Unfortunately, the four biomarkers of interest were not available for all participants, due to limited amount of plasma to analyze for some individuals. We tried to circumvent this aspect by first selecting the plasma markers most related to conversion to AD, which allowed us to conduct further analyses in the full sample, in which p-tau217 level was measured in all participants. Still, the limited plasma quantity precluded us from measuring other p-tau isoforms or Aβ42 and Aβ40 using the most accurate mass spectrometry-based methods (which are superior to plasma Aβ immunoassays used here) [36]. Future studies should evaluate if combing p-tau isoforms with Aβ42/Aβ40 measured using mass spectrometry-based methods would offer improved performance in different stages of AD. Only plasma and no CSF was available in this sample; therefore, we were not able to test how well plasma p-tau levels reflect CSF level. Still, we hypothesize that CSF p-tau217 would have been a key marker related to progression to AD [37]. Given the somewhat small sample size, we also aimed to restrict the number of variables included in logistic regression models and opted for a global score of cognition instead of multiple neuropsychological tests. With memory and executive function being both important cognitive domains to predict AD dementia in the previous large-scale study [1], we derived a modified PACC (we were missing the Free and Cued Selective Reminding Test included in the original version), analogous to the PACC5, which encompassed both domains and is widely used [15, 16]. However, we acknowledge that the Free and Cued Selective Reminding Test might have provided sensitive memory measure to the composite score. Ten participants had missing *APOE*4 genotype, but we replicated the main results when restricting the variables of interest to p-tau217, cognition, and hippocampal volume. We should also mention the current study focused on determining conversion to AD dementia where all converters had brain amyloidosis, while the outcome of the original clinical trial was probable AD, relying on the clinical status from the clinical adjudication committee. Lastly, all participants were categorized as amnestic MCI, and thus generalization of the results to more diverse MCI patients should be determined.

## Conclusions

In MCI patients, plasma p-tau217 was the biomarker most associated with risk of conversion to AD dementia within 3 years. Combining p-tau217 with a few commonly used markers of AD improved the discrimination between those who progressed to AD dementia or not. Aiming for a balance in terms of model fit, parsimony and easily accessible measures, plasma p-tau217, and a score of global cognition were the best markers to predict future decline in this cohort.

## Data Availability

The dataset used for the current study can be available from the corresponding author on reasonable request.

## Abbreviations

Aβ: Beta-amyloid
AD: Alzheimer’s disease
AICc: Corrected Akaike information criteria
APOE: Apolipoprotein
AUC: Area under the curve
CI: Confidence interval
CSF: Cerebrospinal fluid
GFAP: Glial fibrillary acidic protein
MCI: Mild cognitive impairment
mPACC: Modified Preclinical Alzheimer’s Cognitive Composite
NfL: Neurofilament light
PET: Positron emission tomography
p-tau: Phosphorylated tau
